# Evaluation of and Current Trends in the Management of Gastrointestinal Stromal Tumors: A Systematic Review

**DOI:** 10.7759/cureus.26848

**Published:** 2022-07-14

**Authors:** Hadia Arzoun, Mirra Srinivasan, Mona Adam, Siji S Thomas, Amber Kuta, Stephanie Sandoval

**Affiliations:** 1 Internal Medicine, St. Bernards Medical Center, Jonesboro, USA; 2 Internal Medicine, California Institute of Behavioral Neurosciences & Psychology, Fairfield, USA; 3 Internal Medicine, St. Bernards Medical Center, Jonesboro , USA

**Keywords:** current research, drug therapy, treatment, evaluation, gastrointestinal stromal tumors

## Abstract

Gastrointestinal stromal tumors (GISTs) are soft-tissue sarcomas that can occur anywhere in the digestive tract, with the stomach and small intestine being the most common locations. Because no imaging modalities diagnose GIST unequivocally, histological and immunohistochemical confirmation is usually required. Most GISTs are discovered by chance; hence, determining this condition's actual frequency can be challenging. Since diagnosing the tumor could be difficult, including GIST in the differential diagnosis is crucial. The objective of this review is to explore the multiple treatment options for this tumor and provide clinicians with more information on the evolving treatment modalities, which in the future could be a possible solution to cure GIST ultimately. After exploring several studies, the authors conclude that early detection is critical since the treatment depends on the tumor size, mitotic rate, and location. Medical management using targeted therapy approved by the United States Food and Drug Administration (FDA) include tyrosine kinase inhibitors such as imatinib, sunitinib, and regorafenib. Surgical resection of the tumor is also done in cases with localized tumors. Standard chemotherapy and radiotherapy are not commonly used to treat GIST patients. However, radiotherapy may be used as a palliative therapy to ease pain (such as bone pain) or control bleeding. Additional research is needed to establish potential therapeutic targets that will result in higher and longer-term response rates.

## Introduction and background

Gastrointestinal stromal tumors (GIST) are a rare type of malignancy, representing about 2% of all gastrointestinal (GI) cancers [1]. These tumors may be challenging to diagnose in the outpatient setting as many patients exhibit few or nonspecific symptoms, such as abdominal pain or discomfort [2]. In some cases, GIST is associated with gastrointestinal (GI) bleeding, either overt or occult, a sign that necessitates prompt treatment [1]. Gastrointestinal stromal tumors originate in the interstitial cells of Cajal (ICC). These cells are pacemaker cells, as they generate electrical waves in the smooth muscles of the GI. The electrical signal produced by ICC causes four sequential events in smooth muscle cells to occur, including depolarization, activation of voltage-dependent calcium channels, the entry of calcium into the muscle cells, and muscle contraction [3]. These contractions form the basis for peristalsis in the GI tract. Gastrointestinal stromal tumors are most often due to gain-of-function mutations in the CD117 antigen (C-KIT) [4]. The C-KIT gene codes for a tyrosine kinase growth factor receptor for stem cell factor (SCF), a hematopoietic cytokine that plays a vital role in the survival and growth of hematopoietic stem cells and gut motility [5,6]. The most common mutation associated with GIST in the C-KIT gene causes the receptor to become activated without the presence of its ligand, SCF. This type of mutation is typically acquired during a person's lifetime rather than during embryonic development, although the mutation can be inherited. As a result of this mutation, signaling pathways controlled by C-KIT are constantly 'turned on,' causing increased proliferation of ICCs and GIST formation [7].

Overall, the incidence of GIST is low, at 1-2% of all GI tumors. Most GISTs are found in the stomach (56%), followed by the small intestine, colon, rectum, and esophagus. Up to one-third of these tumors become malignant, indicating that most of these tumors are considered benign. Malignant tumors greater than three cm in size with high mitotic rates, particularly in the duodenum, jejunum, ileum, or rectum, are more likely to metastasize [8]. The purpose of this review article is to throw light on the treatment options currently available for GIST. The included studies state that small, non-bleeding tumors may be treated with a watchful-waiting strategy, although moderate to large tumors, bleeding or not, require surgery. Imatinib has also been prescribed as an adjunctive therapy; however, patients who may be refractory to these drugs may benefit from other tyrosine kinase inhibitors such as sunitinib or regorafenib [2,4,8-13].

Methodology

**Figure 1:**
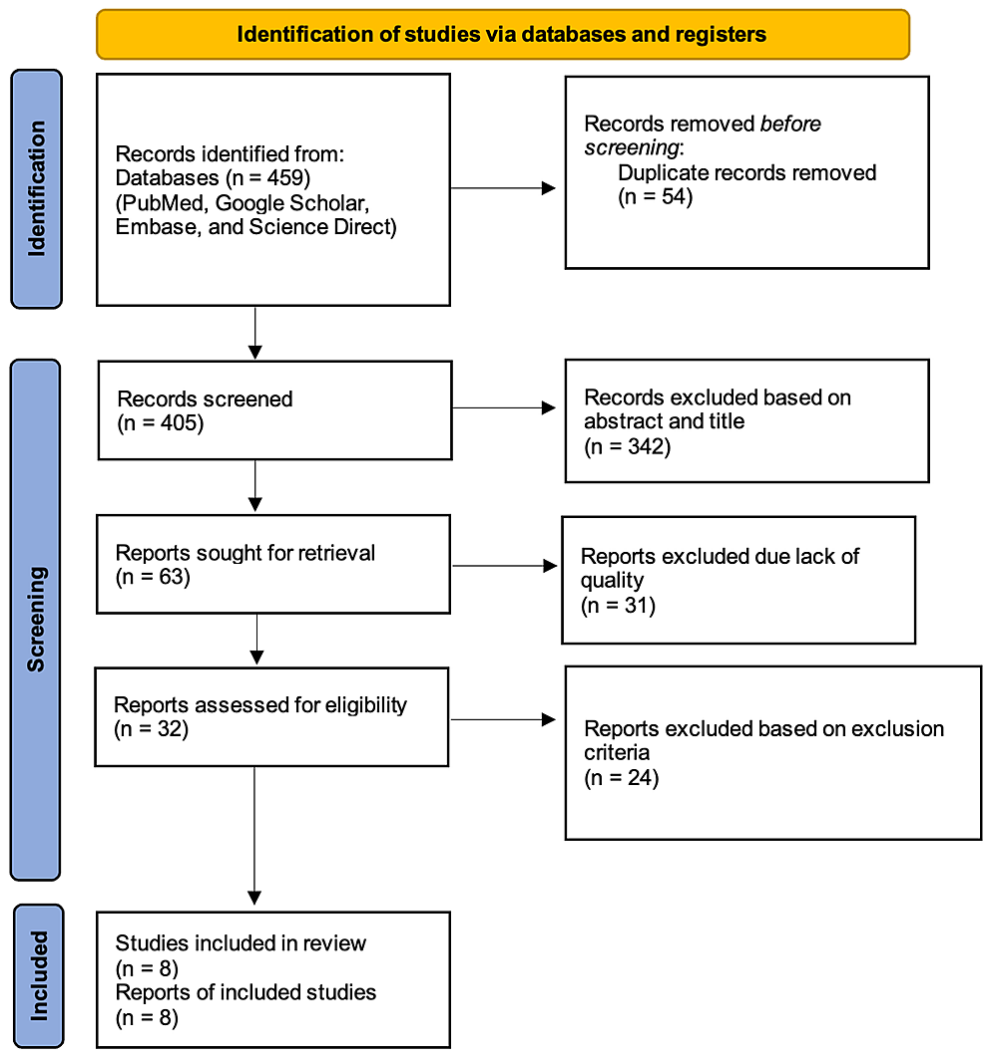
PRISMA Flow Chart 2020 PRISMA: Preferred Reporting Items for Systematic Reviews and Meta-Analysis

This systematic review follows the Preferred Reporting Items for Systematic Reviews and Meta-Analysis (PRISMA) guidelines and principles 2020 [14]. The authors searched the following databases, namely PubMed, Google Scholar, and Science Direct. This study's inclusion criteria comprised reports from the past seven years in the English language with full-text articles. The study designs analyzed included case reports, case series, and review articles. Studies prior to 2015, non-English language reports, non-full-text, and only abstract reports were excluded. Upon exploring the databases mentioned above, a total of 459 articles were sought using the keywords mentioned below. Fifty-four were duplicates; hence they were removed. Four hundred five articles were screened for relevance, 342 were removed due to irrelevant titles and abstracts, and out of 63 reports that were retrieved, 31 were excluded due to lack of quality. The quality assessment was done by two authors independent of each other. In cases where the authors had a difference of opinion, a third author sorted out the differences to attain a mutual agreement. Upon assessing 32 reports for eligibility, 24 reports were omitted based on the exclusion criteria. A final of eight studies were included as eligible reports for this systematic review. The search strategy and the selection process of the final reports are depicted in the form of PRISMA flow chart 2020 in Figure 1.

Keywords

The regular keywords used were:* *Gastrointestinal Stromal Tumors; evaluation; treatment; drug therapy; current research.

The MeSH keywords used were:* *Gastrointestinal stromal tumor OR (( "Gastrointestinal Stromal Tumors/diagnosis"[Mesh] OR "Gastrointestinal Stromal Tumors/drug therapy"[Mesh] OR "Gastrointestinal Stromal Tumors/radiotherapy"[Mesh] OR "Gastrointestinal Stromal Tumors/therapy"[Mesh] ) AND Treatment AND ( "Therapeutics/drug therapy"[Mesh] OR "Therapeutics/pharmacology"[Mesh] OR "Therapeutics/surgery"[Mesh] OR "Therapeutics/therapy"[Mesh] ).

Quality assessment

Table 1 summarizes the individual characteristics of the final included reports [2,4,8-13].

**Table 1:** Quality Assessment of the Included Reports JBI: Joanna Briggs Institute; NHLBI: National Heart, Lung, and Blood Institute; SANRA: Scale for The Quality Assessment of Narrative Review Articles

Author	Year	Type of Study Design	Quality Appraisal Tool Used	Quality Rating (Good, Fair, Poor)
Koumarianou et al. [4]	2015	Case series	NHLBI quality assessment for case series	Good
Giestas et al. [2]	2016	Case report	JBI checklist for case reports	Good
Mulet-Margalef and Garcia-del-Muro [9]	2016	Review article	SANRA checklist	Good
Liu et al. [10]	2018	Review article	SANRA checklist	Good
Parab et al. [8]	2019	Review article	SANRA checklist	Good
Napolitano and Vincenzi [11]	2019	Review article	SANRA checklist	Good
Ahmed [12]	2020	Review article	SANRA checklist	Good
Kelly et al. [13]	2021	Review article	SANRA checklist	Good

Table 2 briefly describes the key points of each report analyzed in this review [2,4,8-13].

**Table 2:** Summary of the Included Reports GIST: gastrointestinal stromal tumor; MDT: multidisciplinary team; EUS: endoscopic ultrasound-guided; TKIs: tyrosine kinase inhibitors; PDGFRA: platelet-derived growth factor receptor alpha

Author	Proposed Treatment	Key Points of the Treatment	Conclusion
Koumarianou et al. [4]	Adjuvant Imatinib	Focused on prolongation of adjuvant imatinib treatment from one to three years.	Extending the adjuvant imatinib course may be beneficial and reduce the risk of recurrence in GIST.
Giestas et al. [2]	Surgery	A laparotomy was performed on the patient, which revealed a jejunojejunal intussusception caused by a jejunal tumor. The lesion was resected, and a GIST tumor was diagnosed on a pathological investigation. The patient was discharged on the seventh postoperative day, and there were no clinical or radiologic signs of recurrence during the follow-up visits.	A rare case of jejunojejunal intussusception induced by a GIST initially manifested as long-term gastrointestinal hemorrhage successfully treated through surgical resection.
Mulet-Margalef and Garcia-del-Muro [9]	Sunitinib	Sunitinib should be taken at a dose of 50 mg per day for four weeks on and two weeks off.	Sunitinib appears to work better in GISTs with KIT mutations at exon nine and in so-called wild-type GISTs.
Liu et al. [10]	Multidisciplinary management of GIST improves both prognosis and quality of life.	The prognosis of GISTs has improved dramatically as tyrosinase inhibitors, such as imatinib, have been more widely available.	MDT and translational medicine may help patients with late-stage tumors that can't be addressed with surgery or who can't take traditional drugs.
Parab et al. [8]	Surgery, Targeted drug therapy, and radiofrequency ablation	Primary low-risk tumors should be resected. High-risk primary or metastatic cancers should be resected with adjuvant imatinib 400 mg daily for one year or neoadjuvant imatinib 400 mg daily followed by surgical resection if the tumor is unresectable. Exon 9, 13, and 14 mutations require sunitinib, whereas exon 17 mutations require ponatinib, and severely resistant cancers require regorafenib. Serial abdominal CT scans should be used to check for recurrence of high-risk cancers. When surgery is not an option, radiofrequency ablation has proven successful. Ipilimumab, nivolumab, and endoscopic ultrasound alcohol ablation are newer therapies that have demonstrated significant results.	The grading of the tumor determines the treatment modality, whether be surgical or medical management. Newer therapies such as ipilimumab, nivolumab, and EUS alcohol ablation are ongoing research.
Napolitano and Vincenzi [11]	TKIs	TKIs are beneficial in GIST; however, resistance occurs through secondary KIT mutations.	To slow the progression of polyclonal imatinib resistance in GIST, therapeutic combinations of TKIs with complementary efficacy against resistant mutations may be beneficial.
Ahmed [12]	Multidisciplinary management	Large GISTs, unresectable GISTs, and metastatic GISTs all benefit significantly from tyrosine kinase inhibitors.	Radiotherapy, chemotherapy, hepatic artery embolization, chemoembolization, and radiofrequency ablation are additional treatment options for metastatic GISTs.
Kelly et al. [13]	Multidisciplinary management	Sunitinib, regorafenib, ripretinib, and avapritinib for advanced PDGFRA D842V mutant GIST are now used as second-, third-, and fourth-line TKIs.	Surgical resection, radiation treatment, and local radiological interventional alternatives may be suitable additions to TKIs, but they must be carefully chosen.

## Review

The following discussion summarizes the current literature about GIST, with and without bleeding, evaluation, and treatment. After a brief presentation of background information, the authors have also focused on assessment in the outpatient setting, including signs and symptoms and diagnostic strategies, and treatment, including surgery and pharmacological options. A brief note on the study's limitations is also mentioned at the end of this section.

Gastrointestinal bleeding can occur in conjunction with GISTs. This common but serious complication occurs in up to 40% of patients [10]. Several reasons exist to explain GI bleeding in patients with GISTs. These include destruction of the mucosa by the tumor, tumor invasion of blood vessels leading to vascular rupture, tumor necrosis, GI peristalsis, and fecal transmission. Tumors in the small intestine tend to bleed more than those found in the stomach. A patient's prognosis with bleeding is typically poor, and bleeding may be a risk factor for recurrent malignancy [10].

Signs and symptoms

The clinical presentation of GIST can vary depending on their size and location and whether mucosal ulceration is present. The most common symptoms and signs are abdominal pain and GI bleeding, although the bleeding is typically neither significant nor persistent [2]. Additional symptoms may include nausea, vomiting, abdominal distention, early satiety, and rarely a palpable abdominal mass. If the tumor is large, the patient may experience dysphagia, jaundice, or constipation, depending on the specific location of the mass [8]. Adults may demonstrate a different clinical presentation than children and adolescents. Adult patients typically present with overt or occult bleeding. Bleeding in the small intestine occurs in 28% of cases, and gastric bleeding can occur in up to 50% of cases. Other symptoms include abdominal discomfort, acute abdomen, and an asymptomatic abdominal mass. Most children and youth with GIST are asymptomatic, and the tumors are discovered incidentally. However, some tumors may grow large enough to cause abdominal pain or GI obstruction or may rupture, causing GI bleeding or bowel perforation [1].

All patients who present with suspected GISTs, as well as abdominal pain, should receive a detailed abdominal examination. These tumors may present as palpable masses. The tumor may have ruptured if the patient exhibits symptoms of acute abdomen, which include abrupt onset of pain, nausea or vomiting, constipation, rebound tenderness, abdominal guarding, absence of bowel sounds, and loss of dullness in the liver upon percussion. In that case, bleeding, a blockage, or perforation may be present [1]. However, given that these tumors are relatively rare and the symptoms may be nonspecific or nonexistent, it can be difficult to diagnose GISTs based only on clinical presentation. While imaging plays a crucial role in diagnosis, an accurate diagnosis is often only possible after surgery and histological examination [2]. 

Diagnostic imaging

Gastrointestinal stromal tumors can be identified using computed tomography (CT), magnetic resonance imaging (MRI), and abdominal ultrasound [1,8]. Using both oral and intravenous contrast, contrast-enhanced CT is the preferred imaging method to determine if an abdominal mass is a GIST. A GIST appears as a solid and smooth mass with bright enhancement on a CT scan. Large tumors exceeding 15cm may appear complex in morphology. This imaging strategy can also provide information about invasion by the mass into nearby structures. However, it may be difficult to identify the primary location of a large mass using CT [1]. A second imaging strategy is an MRI of the abdomen and pelvis. Though not the preferred method, MRI has the advantage of no radiation exposure for patients. This form of imaging may be more helpful than CT in evaluating primary rectal GISTs, liver metastases, bleeding, and tumor necrosis [8]. Small tumors detected using MRI appear round and symmetric, while larger ones may be asymmetrical and contain lobules [1]. Abdominal ultrasound is also not a primary means of visualizing GISTs. However, this strategy may be helpful when the tumor size exceeds five cm. Issues that can impact the reliability of ultrasound include air in the bowel, ulceration, or necrosis [8]. 

Diagnostic procedures

Additional diagnostic procedures may include upper endoscopy, colonoscopy, or rectal ultrasound. Upper endoscopy with ultrasound helps characterize upper GI tumors involving the stomach, small intestine, or esophagus. The advantage of adding endoscopic ultrasound to the procedure is identifying the tissue layer of origin. Most tumors originate in the fourth layer of the GI tract, the muscularis propia, while smaller tumors typically originate in the second layer, the muscularis mucosa [1]. Colonoscopy is useful for identifying masses in the colon, rectum, and anus. Small masses can be biopsied during a colonoscopy, but larger masses are more challenging. Finally, rectal endoscopic ultrasound may help understand the anatomy of the mass prior to a needle biopsy but is typically not helpful in the staging of tumors [1].

Histopathology

Histopathology and immunochemistry are useful in confirming a GIST diagnosis. These tumors assume three morphologies, including spindle, epithelioid, or mixed type. Cells with a spindle morphology, including their nuclei, appear elongated. Epithelioid cells are rounded with a centrally-located nucleus. Immunochemistry includes staining the biopsied cells for the presence of CD117, which is positive for the receptor in 95% of cases [8].

Staging and grading

Accurate staging and grading of GISTs help determine the potential effectiveness of treatments [8]. According to one research, there are at least 16 types of classification systems. These systems can be designated as categorical, in which patients are stratified into risk groups, or continuous, in which an individualized risk assessment is performed [15]. The most common variables considered in this collection of classification systems are tumor size, location, and mitotic count. The latter of these variables refers to the number of cells in a sample undergoing cell division [15]. Of the existing systems, the most widely accepted scales are those developed by the French Federation of Cancer Centers Sarcoma Group (FNCLCC) and the National Cancer Institute (NCI). However, the FNCLCC system is more precise and reliable in predicting outcomes than the NCI system and is therefore recommended for use by the American Joint Committee on Cancer and the College of American Pathologists [16].

The FNCLCC grading system considers three tumor characteristics in the staging process, including tumor differentiation, mitotic count, and tumor necrosis [16]. The tumor differentiation scale consists of three scores (1-3), including tumors that closely resemble normal tissue (score 1), tumors confirmed through histologic typing (score 2), and tumors that are undifferentiated or of uncertain differentiation (score 3). The mitotic count also ranges from a score of one to three, with categories of 0-9, 10-19, or more than 20 mitotic cells per 10 contiguous high-powered fields. Tumor necrosis confirmed through gross examination or histologically is assigned a score ranging from zero to two. Bleeding is not considered in the assessment of necrosis. Necrosis is categorized as non-existent (score 0), <50% tumor necrosis (score 1), or >50% tumor necrosis (score 3). The scores from these three variables are then summed to reveal the histological grade. A total score of two or three is considered to be a Grade 1 (low grade) GIST, while a score of four or five is a Grade 2 (intermediate grade). A score of six, seven, or eight is a Grade 3 tumor (high grade) [16].

The National Institutes of Health (NIH) developed an additional grading system that classifies patients according to risk category based on tumor size and mitotic count per 50 contiguous high-power fields [12]. Patients at very low-risk exhibit tumors less than two cm with mitotic counts of less than five. Patients at low risk demonstrate the same level of mitotic counts but have tumors slightly larger in size, ranging from two-five cm. Intermediate-risk patients exhibit tumors less than 5 cm in size but with mitotic counts between 6-10/50 high power fields or larger tumor size (five-10 cm) with mitotic counts less than 5/50 high power fields. Finally, patients categorized at high-risk exhibit tumors exceeding five cm in size and mitotic rates exceeding 5/50 high power fields, tumors larger than 10 cm with any mitotic rate, or tumors of any size with a mitotic rate exceeding 10/50 high power fields. Using this classification system, 44% of GISTs are considered high-risk, 24% are intermediate risk, and 32% are low or very low risk [12].

Treatment modalities

Surgery

Surgery is the first line of treatment for GISTs with and without bleeding [10]. If the tumor is small, such as less than two cm, and not associated with bleeding, then a watchful-waiting approach may be used. For larger tumors and those associated with hemorrhage, traditional surgical methods are typically used to resect the tumor [10]. Some tumors may be treated using endoscopy. However, while endoscopy may have a short-term benefit, long-term results are still unknown. Additionally, endoscopy has a lower rate of complete tumor excision than conventional surgical techniques. Furthermore, endoscopy can increase the potential for GI tract perforation and tumor rupture, and bleeding. Laparoscopy, like endoscopy, is not a preferred surgical technique. Laparoscopy is recommended for stomach tumors less than five cm in diameter, but the long-term effects of this technique on tumor prognosis remain unclear [10].

Medical Management

The first line of pharmacological therapy for GIST is imatinib. First approved in 2002, this small molecule drug inhibits three different receptor tyrosine kinases. As mentioned previously, C-KIT is a tyrosine kinase receptor, which means that it participates in signaling pathways by phosphorylating other proteins in the pathway. By inhibiting the function of this tyrosine kinase, the rogue signaling pathway in GIST cells may be shut down, thus preventing tumor cell proliferation. Initial clinical trials with this drug indicated that 53.7% of participants achieved at least a partial response. The mean time to respond was three months, and the optimal dose was 400 mg/day. Adverse effects include edema, nausea, diarrhea, myalgias, fatigue, dermatitis, headache, and abdominal pain [13]. For 10% of patients taking this drug, the response is sustained for at least ten years [11].

Clinical guidelines indicated that patients should be treated with imatinib for one to three years in order to reduce the risk of relapse. The average time to tumor progression after treatment initiation is two to two and a half years [10]. When progression does occur, the daily dose can be increased to 800 mg. A higher dose may also be effective for patients with mutations in exon 9 of the CD117 gene, although the majority of these individuals do not respond to imatinib [10]. In the case of this specific mutation, guidelines recommend starting doses between 600-800 mg/day. However, the side effects associated with the higher doses may counteract the drug benefits resulting in no net survival benefit [10].

Not all patients will respond to imatinib, as this drug is most effective against a particular C-kit mutation. The majority of patients with GIST, 70%, possess a mutation in exon 11 of the CD117 gene. Most patients, 85%, with this mutation will respond to imatinib [8]. However, lower percentages of patients with mutations in other CD117 exons respond to the drug. Furthermore, the CD117 gene is not the only gene whose mutations lead to GIST. Mutations in the gene coding for platelet-derived growth factor receptor alpha (PDGFRA) affect 10% of patients with GIST and are typically associated with stomach tumors [13]. Patients with mutations in the PDGFRA gene typically do not respond well to imatinib [8]. This variation in drug response due to specific mutations underscores the need for genetic analysis of tumors in GIST patients.

Additional drugs are available that target cells with mutations other than those found in exon 11 of the CD117 gene. Sunitinib can inhibit cells with mutations in other CD117 exons, as well as mutations found in PDGFRA [9]. The recommended starting dose for sunitinib is 50 mg/day for 28 days with seven-day breaks between cycles. This particular dosing schedule is beneficial to a greater percentage of patients than higher or lower doses, although dosages can be adjusted and optimized based on individual characteristics [17].

A third drug with the potential to treat imatinib-resistant GISTs is regorafenib, which targets cells with various mutations in CD117 and/or PDGFRA. This kinase inhibitor may produce at least a partial response in 79% of patients for 16 weeks or more. The recommended starting dose is 160 mg/day for three weeks before taking a one-week break [13]. Another possible treatment currently under investigation is the simultaneous use of sunitinib and regorafenib at lower doses, at 37.5 mg/day and 120 mg/day, respectively. Clinical trials may be the best option for patients who do not respond to any of these treatments [13].

Limitations

This review mainly focuses on the current therapeutic modalities of GIST and does not look back beyond 2015. The main objective is to show light on the various treatment options, and other aspects of the tumor are not discussed in detail. The study includes only case reports, case series, and review articles.

## Conclusions

Gastrointestinal stromal tumors, which represent a small percentage of overall GI tumors, can be challenging to identify in the outpatient clinical setting. Symptoms associated with these tumors, which typically include abdominal pain or discomfort, can also suggest other diagnoses. In order to confirm a diagnosis of GIST, contrast CT is used to visualize the mass along with histopathology and immunochemistry. Small non-hemorrhaging tumors may initially warrant a watchful-waiting approach, while moderate to large tumors with or without bleeding require surgery. Adjunctive treatment consists of imatinib, the first line of therapy for GIST. Patients who do not respond to this drug due to the presence of certain genetic mutations may benefit from other tyrosine kinase inhibitors such as sunitinib or regorafenib. Current research concludes that a multidisciplinary treatment approach will not only improve the quality of a patient’s life but will also steer towards a better prognosis and improve survival. However, given that these biological therapies may not be effective at halting disease progression in the long- term, further research is needed to uncover newer treatment approaches that yield improved and longer-term response rates.
